# Additive Manufacturing of Oral Tablets: Technologies, Materials and Printed Tablets

**DOI:** 10.3390/pharmaceutics13020156

**Published:** 2021-01-25

**Authors:** Alperen Abaci, Christina Gedeon, Anna Kuna, Murat Guvendiren

**Affiliations:** 1 Otto H. York Department of Chemical and Materials Engineering, New Jersey Institute of Technology, Newark, NJ 07102, USA; aa2848@njit.edu (A.A.); cg452@njit.edu (C.G.); ak794@njit.edu (A.K.); 2 Department of Biomedical Engineering, New Jersey Institute of Technology, Newark, NJ 07102, USA

**Keywords:** 3D printing, polymer, hydrogel, pharmaceutical, precision medicine, drug delivery

## Abstract

Additive manufacturing (AM), also known as three-dimensional (3D) printing, enables fabrication of custom-designed and personalized 3D constructs with high complexity in shape and composition. AM has a strong potential to fabricate oral tablets with enhanced customization and complexity as compared to tablets manufactured using conventional approaches. Despite these advantages, AM has not yet become the mainstream manufacturing approach for fabrication of oral solid dosage forms mainly due to limitations of AM technologies and lack of diverse printable drug formulations. In this review, AM of oral tablets are summarized with respect to AM technology. A detailed review of AM methods and materials used for the AM of oral tablets is presented. This article also reviews the challenges in AM of pharmaceutical formulations and potential strategies to overcome these challenges.

## 1. Introduction

The majority of the drugs are administered orally in the form of a solid dosage form. Oral tablets offer dose precision, chemical and microbial stability, controlled drug release profiles, and ease of administration [1,2,3]. Oral tables can easily be carried by the patient, making them available when needed (on-demand administration). Despite these advantages, conventional tablet manufacturing methods include time- and labor consuming procedures, including a multitude of steps: (i) bulk powder material handling and mixing of excipients and active pharmaceutical ingredients (APIs), (ii) powder processing such as compression and wet or dry granulation, (iii) tablet compression and testing, (iv) tablet relaxation, (v) tablet coating, and (vi) tablet collection and handling [4,5,6]. Continuous (oral solid dosage) manufacturing (CM) enabled full integration of bulk powder handling to final tablet product, yet CM offers limited dose flexibility and tablet customizability [7,8]. Additive manufacturing (AM) technology enables the fabrication of custom-designed oral tablets with high architectural, structural, and compositional complexity [9,10,11], and could potentially lead to a paradigm shift in tablet manufacturing from a one fit all approach to personalized medicine.

Additive manufacturing (AM), also known as 3D printing, is a layer-by-layer fabrication method utilizing a printable material, or ink, to create a 3D object from a digital image developed via computer-aided design (CAD). Multi-material AM approaches enable precise positioning of a multitude of distinct materials or combination of materials to create compositional complexity, including multi-phasic 3D constructs with each phase constituting a distinct composition, as well as constructs with compositional gradients. Note that the printability of a material is directly determined by the AM technology, which also dictates the form of the ink, including filament, solution, melt, slurry, or powder [12]. Overall, AM could offer significant design flexibility in oral tablet manufacturing by enabling custom-designed tablets matching the target patient for personalized medicine. Tablets could be personalized by tailoring the target dose for single APIs or combination of APIs and their release profile according to the patient’s age and weight, and/or the severity of the disease [13]. In addition, AM could potentially be instrumental for early-phase drug development scenarios including evaluation of oral dosage forms for preclinical studies (including dose flexibility), exploration of custom designs (shape, porosity, and composition), and on-demand manufacturing (at clinical site) as well as an overall reduction in utilization of resources. Despite these potential advantages, there is a big gap in additively manufactured oral tablets in the market after the first 3D-printed tablet in the market, Spritam^®^, was approved by the United States Food and Drug Administration (FDA) in 2016.

In this review, we summarize AM technologies used in oral tablet fabrication, widely used materials in additively manufactured oral tablet formulations, and tablets printed for each AM technology. We also present the challenges in AM of pharmaceutical formulations and potential strategies to overcome these challenges.

## 2. AM Technologies

**Figure 1:**
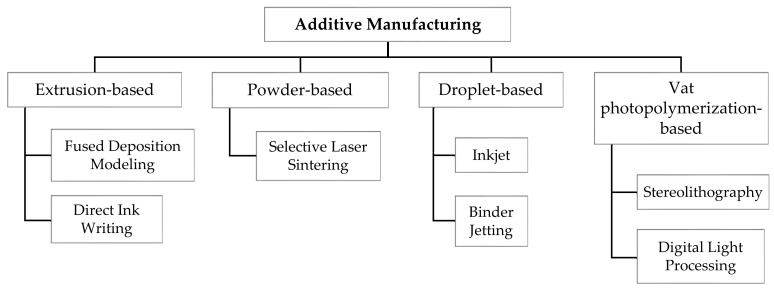
The additive manufacturing technologies used in oral tablet fabrication.

In this review, the AM technologies used for oral tablet printing are classified under four main groups: extrusion-based, vat photopolymerization-based, droplet-based, and powder-based printing (Figure 1).

Extrusion-based printing includes filament printing, commonly known under the trademark name fused deposition modeling (FDM), and direct ink writing (DIW). In extrusion-based printing, the ink, i.e., the printable material in the form of a viscous melt or liquid (or slurry), is extruded through a nozzle forming individual struts (or lines) that solidify onto the build substrate. The nozzle follows a custom-designed line path determined by the g-code (computer-aided design) to form a 3D object in a layer-by-layer manner. For FDM, the ink is a thermoplastic solid filament. This filament is pulled into a hot nozzle and extruded as a melt. For DIW, the ink is a viscous melt, liquid or slurry (30–6 × 10^7^ mPa.s). When polymer solutions are used as an ink, low boiling point solvents (such as dichloromethane (DCM) or tetrahydrofuran (THF)) are preferred to ensure rapid evaporation of the extruded ink to form a solid polymer [14].

Powder-based printing technology includes selective laser sintering (SLS), which allows printing of powders (polymers, ceramics, and metals, as well as their composites). In SLS, a laser beam moves over the powder bath raising the temperature of the powder particles on its path to sinter or fuse the particles spatially. Once a single layer is formed, the build platform moves down and a fresh layer of powder is applied from the top, and the process is repeated. The ink is in the form of a fine powder (10 to 100 μm in diameter) with good flow properties within the bed system. SLS machines usually require a large amount of powder, and they are not readily available [15,16].

Droplet-based printing technologies include inkjet printing and binder jetting (BJ). The ink is a low viscosity solution (viscosity below 10 cP (mPa.s)), which is ejected as an individual droplet (25–100 μm in diameter; 1–100 picoliters) [17]. In inkjet printing, the droplet is required to be placed on the print substrate and coalesce with the adjacent droplets to form a solid line. Similar to extrusion-based printing, the nozzle follows a custom-designed line path to form a 3D object in a layer-by-layer manner. The ink is usually exposed to high shear rates (0.1–1 × 10^6^ s^−1^), and the inks should have a surface tension in the range of 28–350 mN·m^−1^ to ensure proper ejection from the nozzle and shape of the droplet on the substrate [18,19,20,21]. Similar to the DIW process, the liquid-to-solid transformation is crucial to determine the final shape of the printed structure. When the ink is printed directly on a powder bath surface, the process is referred to as BJ. Note that BJ could also be considered as powder-based printing. In BJ, ink is a binding solution which binds the powder particles together. In this process, the ink droplets bind the powders to form a layer. The powder platform moves down and a fresh powder layer is brought on top, and the process repeats itself [22].

In vat photopolymerization-based printing, the ink is a photocurable viscous liquid (i.e., a prepolymer, macromer, or a monomer), and photocuring refers to light-induced polymerization (photopolymerization) and/or crosslinking (photocrosslinking). In traditional stereolithography (SLA) printing, a beam of light (e.g., UV or laser) moves over the vat and cures ink spatially. The beam follows a pattern (defined by the g-code) to create a layer. After each layer is completed, the build stage moves down into the vat. Recent SLA printers are inverted, thus pulling the printed layer up, which significantly reduces the required depth of the vat, and hence the amount of required ink (~2500 mL). In direct light processing (DLP) printing, rather than using a single beam of light, a whole print layer is directly projected, leading to curing of a print layer at each exposure. Although this significantly increases the print speed, it may reduce the resolution. Although vat photopolymerization printing has high resolution (the size of a single beam is ~25 microns), it requires extensive postprocessing (discussed below) and has limited ink formulations [23,24].

## 3. Materials for 3D Printing Oral Tablets

### 3.1. Polymers

Polymers are the most versatile category of biomaterials and have a wide range of utilizations in tablet development. Polymers are mainly used as excipients to improve the function and delivery of the APIs, yet polymers can also be designed as APIs (or drugs) [25]. In this review, we will focus on the former case where the polymer has no therapeutic effect. The suitable polymer tablet printing should be (i) biocompatible, (ii) easily cleared from the body (without side effects), (iii) able to deliver the APIs without interacting with them, (vi) amenable to control the release profile of APIs, and (v) printable. The requirements for printability are determined by the selected printing technology as described above. The selection of the polymer is based on the printing technology and the desired release profile. The form of the polymer used is also determined by the printing technology, and this includes filament, powder, paste, hydrogel, solution, or colloidal suspension. Below we summarized the most commonly used polymers for tablet printing.

#### 3.1.1. Cellulose-Based Polymers

Due to their abundance in nature and favorable properties (low-cost, biocompatibility, and biodegradability), cellulose and its derivatives received increased attention in a wide range of applications including pharmaceuticals [26]. For tablet printing, cellulose derivatives are preferred over pure cellulose, as cellulose macromers display very strong hydrogen bonding, affecting their melting and solubility. When heated, cellulose degrades before melting. Cellulose derivatives, such as cellulose acetate [27,28], ethyl cellulose (EC) [29,30], hydroxypropyl cellulose (HPC) [29,31,32,33,34,35], and hydroxypropyl methyl cellulose (HPMC) [29,36,37], have been frequently used to enhance printability and to modulate the drug release profile during dissolution. The most commonly used 3D printing technology for cellulose-based polymers is FDM printing, for which 3D printable filaments containing APIs are fabricated by hot melt extrusion (HME) [38,39]. 3D printable solutions of cellulose-based polymers are also prepared for DIW to print tablets at room temperature [36,37]. Characteristics of cellulose derivatives, such as molecular weight, can impact their use in the polymer formulation. For example, low molecular weight HPC can be utilized as a binder, while high molecular weight HPC can be used as a controlled release matrix [40].

#### 3.1.2. Poly (Vinyl Alcohol)

Poly (vinyl alcohol) (PVA) is a water-soluble polymer that is produced by partial or complete hydrolysis (generally above 80%) of polyvinyl acetate through the removal of the acetate group. The degree of hydrolysis determines the characteristics, such as molecular weight, water solubility, and mechanical properties, of the PVA [41]. For example, the melting point of PVA is higher for fully hydrolyzed polymers and ranges from 180 to 220 °C. Additionally, the higher the degree of hydrolysis, the lower the molecular weight and the higher the solubility in water [42]. Because of its biocompatibility, non-toxicity, water solubility, and good mechanical and swelling properties, PVA has gained attention as an excipient to oral solid dosages [43]. It is poorly absorbed in the gastrointestinal tract and easily eliminated from the body; however, the molecular weight of PVA affects this behavior. PVA is a colorless, tasteless, and odorless thermoplastic. It is Generally Recognized as Safe (GRAS) by the FDA since it is inert, stable, and proven to not show any adverse effects when administered in the body. It is also included in the FDA inactive ingredients database [44]. PVA should not be combined with a compound having secondary hydroxy groups because it may undergo esterification and result in unwanted changes to the structure of the mixture. Due to its favorable characteristics, PVA is mainly utilized with an FDM printer to fabricate oral tablets in the filament form, and APIs are incorporated with PVA either using HME [45,46,47,48,49,50,51] or by soaking in a drug solution [52,53,54,55]. Besides the production of oral solid dosages, PVA is also widely used in the pharmaceutical industry to make transdermal patches [56,57], topical delivery systems [58,59], and mucoadhesive and viscosity enhancers for ocular delivery [60].

#### 3.1.3. Eudragit

Eudragit is a methacrylic polymer that was first introduced in the 1950s for enteric coatings. It is prepared by polymerization of acrylic and methacrylic acids and their esters [61]. These are amorphous polymers with a T_g_ (glass transition temperature) usually in the range of 91–50 °C. These polymers can achieve a flexible and targeted release profile, such as an immediate or a sustained release, depending on the functional groups incorporated into them. For example, Eudragit S and L can withstand the acidic environment of the stomach, enabling drug to be released in the intestine, whereas Eudragit E dissolves in acidic conditions up to pH = 5 (enabling release in the stomach) [39]. In addition, Eudragit is available in different degrees of solubility [62]. A detailed review focusing on the pharmaceutical significance of Eudragit can be found from Patra et al. [63]. The release of the API depends on the degree of Eudragit used, since its solubility depends on the pH. Eudragit has been used to optimize ophthalmic [64,65], buccal or sublingual [66], enteric [67], oral [68,69], colon [70], vaginal [71], and transdermal [72] delivery. Eudragit derivatives (Eudragit RL, RS, E, and L100-55) [73,74,75,76] are commonly utilized to 3D-print tablets using the FDM printing technology. Although FDM is the most commonly used technology to fabricate tablets with Eudragit, Eudragit in powder form was used for binder jetting technology as well [77,78].

#### 3.1.4. Polyvinylpyrrolidone

Polyvinylpyrrolidone (PVP) is synthesized by free radical polymerization from its monomer N-vinylpyrrolidone. PVP is soluble in water and polar solvents but insoluble in hydrocarbons, and its solubility is determined by the degree of polymerization. The molecular weight of PVP ranges from 2500 to 2,900,000 Daltons [79]. PVP is successfully employed in 3D printing technologies, such as FDM [80,81], DIW [82], and binder jetting [83], to form oral tablets. PVP also acts as a coating agent or a binder for wet granulation due to its good wetting properties [84,85]. In addition, it is widely used for topical delivery by mixing it with iodine to form a complex used for solutions and ointments. It is also added in formulations for parenteral and ophthalmic administrations [86]. PVP is physiologically inert and considered safe to use in pharmaceutical applications [87]. It can be kept under ordinary conditions without undergoing degradation or decomposition. It is only affected if the temperature reaches 150 °C, at which point it darkens and becomes less soluble [44]. Another important storage condition is to prevent PVP from moisture absorption, since it is a hygroscopic polymer.

#### 3.1.5. Polycaprolactone

Polycaprolactone (PCL) is a semi crystalline hydrophobic aliphatic polyester, which can be synthesized via polycondensation of hydroxycarboxylic acids and catalytic ring-opening polymerization of lactones [44]. PCL is not soluble in either water or alcohol. Degradation of PCL in the body can take two to four years, which makes it more suitable for long-time degradation/release devices [42,88]. PCL is GRAS by the FDA [44], which allows it to be utilized as scaffolding for tissue engineering [89,90] and wound dressing [91], as well as drug delivery devices [88,92,93]. Although PCL is not widely used in tablet printing due to its poor solubility, it was utilized with SLS to prepare porous matrix devices [94,95] and with FDM to fabricate model tablets [96].

#### 3.1.6. Carbopol

Carbopol is a high molecular weight, crosslinked synthetic polymer. Carbopol is synthesized in ethyl acetate or an ethyl acetate/cyclohexane mixture. Crosslinking is performed with allyl sucrose or allyl pentaerythritol. The crosslinking degree determines the viscosity of the polymer. Carbopol 971P, a lightly crosslinked formulation, has a viscosity range of 4000 to 11,000 cP (or mPa.s) and Carbopol 974P, a highly crosslinked formulation, has a viscosity range of 29,400 to 39,400 cP [39]. Carbopol can be incorporated in drug-loaded paste formulations for DIW printing. Its function within the printed tablets is generally to provide sustained release of the API [36]. Carbopol can also be used as a binder in drug formulations [97].

#### 3.1.7. Polyethylene Glycol

Polyethylene glycol (PEG) is a water-soluble and a biodegradable polymer, and its derivatives, such as mono- and diacrylates, are widely used in tissue engineering applications. PEG derivatives are generally utilized as photopolymerizable (photocurable) polymers, making them attractive for lithographic applications [98,99,100]. PEG derivatives, such as PEG diacrylate (PEGDA) and PEG dimethacrylate (PEGDMA), were used as photocurable inks to print oral tablets using inkjet printing [101], SLA [102,103,104], and DLP [105,106] technologies. The main mechanism of drug release in tablets fabricated with degradable polymers is diffusion of the drug. Water content and the degree of crosslinking of the polymer have a direct effect on drug release profiles. Martinez et al. fabricated ibuprofen-loaded PEGDA tablets using an SLA printer and showed that increased water content will improve diffusion of ibuprofen and result in faster drug release [104]. Wang et al. showed that, when paracetamol and 4-ASA loaded PEGDA solutions were used in an SLA printer separately, higher PEGDA concentrations showed slower drug release, which indicates that higher degrees of crosslinking make the diffusion of the drug harder [102].

#### 3.1.8. Polymer Blends/Mixtures

In addition to the polymers listed above, polymers can be mixed to optimize the printability of a pharmaceutical formulation [107]. For instance, Ilyés et al. tested the printability of different polymeric blends, such as Kollidon SR (8:2 of PVA:PVP), Affinisol 15LV (modified HPMC with a lower glass transition), and other mixtures, using an FDM printer [108]. Fina et al. used Kollicoat IR (75% PVA and 25% polyethylene glycol) and Eudragit L100-55 (50% methacrylic acid and 50% ethyl acrylate copolymer) to print paracetamol tablets using SLS to show the versatility of an SLS printer in tablet printing [109]. Shi et al. printed tablets using a binder jetting technology. During the printing process, a binder solution containing 2-pyrrolidinone and a CaSO_4_-based powder bed were used. To incorporate the model drug (5-fluorourancil) into the tablet, 5-fluorourancil-loaded coating solutions containing Soluplus (co-polymer of polyvinyl caprolactam, polyvinyl acetate, and PEG) incorporated with or without additional PEG were dropped on the tablets using micropipettes. The authors showed that the dissolution profile could be adjusted by changing drug coating solution compositions (Soluplus and PEG concentrations) and it was possible to fabricate tablets with different formulations/dimensions using powder-based printing [110].

### 3.2. Additives

Since a convenient oral solid dosage cannot be achieved with the APIs alone, additives, or excipients, are incorporated into the pharmaceutical formulations. These have no therapeutic effect and take up the majority of the formulation but play a critical role in the pharmaceutical performance and release profile of the APIs. They improve the processing quality during manufacturing and enhance stability, effectiveness and patient compliance [111,112]. Additives can be either natural or synthetic [113]. An ideal additive needs to be inert, inactive, physically and chemically stable throughout the shelf life of the tablet, and compatible with other additives and APIs. It should also comply with regulatory requirements. When making tablets, additives such as plasticizers, lubricants, disintegrants, binders, and fillers, as well as coating agents, stabilizers, emulsifiers, and viscosity enhancers can be added to the pharmaceutical formulations [114,115].

Although it is not mandatory to incorporate additives in the 3D printing process of oral tablets, some technologies benefit from these additives substantially. Plasticizers, lubricants, binding agents, and fillers are mainly incorporated into pharmaceutical formulations to improve the printing process, while disintegrants are used to optimize the dissolution profile of the API in the pharmaceutical formulation. Plasticizers are generally the main additive used in tablet printing, as they ensure the optimized mechanical and thermal properties of the final product. However, other additives are also important as they make the printing process much smoother. Although the effect of additives is mostly studied with FDM printing, binders are generally of interest in BJ printing of oral tablets. Below we summarize the most important additives that are used for 3D-printed oral tablets.

#### 3.2.1. Plasticizers

Plasticizers are added to polymers or polymeric blends to improve mechanical and thermal properties of the oral tablets [116,117]. They are inert, organic, and non-volatile compounds with low molecular weight. Plasticization can be done either internally by chemically modifying the polymer (by directly altering the backbone chemistry or incorporating/altering pendant chains) or externally by blending the polymer mixture with a plasticizer (without altering the chemistry of the polymer) [118]. External plasticizers are classified into two categories: primary and secondary plasticizers. Primary plasticizers are added to lower the glass transition temperature to enhance flexibility, processability, and distensibility [119]. On the other hand, secondary plasticizers are mainly used in combination with primary plasticizers to enhance the effect of the primary plasticizer [118,120]. The most commonly used plasticizers in the pharmaceutical industry are citrate ester-based plasticizers, such as triethyl citrate (TEC), tributyl citrate, and acetyl triethyl citrate. Aside from these, fatty acid esters, sebacate esters, phthalate esters, glycol derivatives, and vitamin E TPGS (D-α-tocopherol polyethylene glycol 1000 succinate) are common types of plasticizers [121]. TEC is one of the most commonly used plasticizers in tablet printing applications, especially when FDM printing is used [48,73,74].

#### 3.2.2. Lubricants

Lubricants are added in small quantities when mixing dry powders during the ink preparation step before printing or extruding them into 3D printable filaments. The main idea of using lubricants is to ensure the continuity of the tablet printing process, such as by preventing clumping to ensure a homogeneous mixture, to reduce friction, and to improve powder flow [122]. Pharmaceutical lubricants can be classified as glidants, anti-adherents, and die wall lubricants [123]. Glidants improve the flow properties of the powder blends by reducing interparticle friction to prevent insufficient mixing and poor content uniformity. Anti-adherent additives reduce the adhesion and prevent the mixture from sticking onto the processing equipment and printer, such as the powder/ink mixing equipment, melt extruder, print head (for extrusion-based printing), or powder print bath (for powder-based printing). Die wall lubricants reduce the friction between the powder particles and the die wall when compacting powders [122]. The main lubrication mechanisms include hydrodynamic, elastohydrodynamic, and mixed and boundary lubrication [124,125]. Lubricants are essential for robust and successful manufacturing of tables for both conventional and additive manufacturing approaches. A good lubricant should have low shear strength, should not be toxic and should not be affected by the process variables [126]. Lubricants can be either hydrophilic or hydrophobic; however, hydrophobic ones are more frequently used because they are effective at low concentrations. Different kinds of lubricants, such as oleic acid and magnesium stearate, were successfully incorporated in 3D printing of tablets [76,127]. The most commonly used lubricants for conventional manufacturing of tables are talc, silica, magnesium stearate, and stearic acid [115].

#### 3.2.3. Disintegrants

The main role of disintegrants is to accelerate the drug release by enhancing disintegration and dissolution. Disintegrants ensure fragmentation of the tablet into smaller particles upon ingestion to allow the onset of dissolution and absorption. To initiate the disintegration process, they promote moisture penetration into the tablet. In general, disintegrants are hydrophilic, and swelling of the disintegrant initiates the breakup of the tablet [128,129,130,131]. The degree of swelling depends on the chemical structure and the crosslinking of the disintegrant as well as the porosity of the tablet [132]. Furthermore, the performance of the disintegrant mainly depends on the particle size and the moisture content [133]. The most frequently used disintegrants for pharmaceutical formulations are starch, cellulose, and their derivatives [131]. Desai et al. tested rapidly disintegrating tablets incorporating APIs with different solubilities, such as ascorbic acid, aspirin, and ibuprofen, while investigating the effect of different disintegrants, including croscarmellose sodium (CCS), crospovidone (CP), sodium starch glycolate (SSG), and microcrystalline cellulose (MCC), on the dissolution and tablet hardness [134]. Dissolution tests showed that increasing the disintegrant concentration resulted in faster disintegration; however, at some critical concentration, the disintegration time started to increase. The fastest disintegration times for aspirin and ibuprofen tablets were obtained with 8% SSG, 7% CCS, or 8% CP disintegrant concentrations. For ascorbic acid, these values were 6% SSG, 7% CCS, or 6% CP. For aspirin, increasing the disintegrant concentration resulted in lower hardness values, although there was a minima and it started to increase again for MCC, CCS, and SSG. For ibuprofen the relationship was more linear. Increasing CCS, SSG, and MCC content improved the hardness of the tablets, while additional CP content decreased the hardness. Sadia et al. tested different disintegrants to examine their effect on the drug release profiles of the FDM-printed tablets [74]. In their study, disintegrants, including ac-di-sol, primellose, primojel, polyplasdone-XL, and explotab, were used. Their results showed no significant difference between disintegrants when the drug release profiles of the tablets were considered. The authors suggested that this could be due to high polymer concentrations used in the drug formulations, which could lead to coating of the disintegrants during the melt printing process [74].

#### 3.2.4. Binding Agents

Binding agents are used to increase cohesion in the powder mixture, leading to improved hardness and friability. They are either added into the solution or in dry powder form. The three types of binders commonly used in the pharmaceutical industry are natural binders, synthetic binders, and sugars [135]. Natural binders are advantageous because they are abundant (with low cost), biodegradable, and ready for use. Natural binders can also act as a transport medium for the drug to the site of absorption. Some examples include starch, acacia, and gum. Synthetic binders are mixtures of polymers, resins, and oils. Some examples of commonly used polymers include polyvinyl chloride (PVC), HPMC, and methyl cellulose. Sugars include glucose, sucrose, and sorbitol [135]. Polymeric binding agents are hydrophilic, and they increase the wettability of poorly soluble drugs, resulting in improved dissolution. Binders, such as Eudragit L100 [78] and polyvinylpyrollidone K30 [136], are commonly used with BJ technology in tablet printing.

#### 3.2.5. Fillers

Fillers are added to formulations where the API is present in small quantities and is not enough to form a tablet. They increase the volume of the mixture and allow fabrication of an average-size pill. Fillers usually have a weak binding capacity; thus, binders and fillers are used together. Sadia et al. tested the nature of tri-calcium phosphate (TCP) as a filler by preparing ink formulations with a range of polymer (Eudragit EPO) to filler (TCP) ratio to 3D-print tablets using FDM printing. The authors showed that when Eudragit EPO filaments without TCP was used in an FDM printer, fabricated structures were deformable and showed poor features (resolution). However, addition of TCP as a thermostable filler allowed reproducible tablet printing with improved structural features. Optimum TCP content was found to be 20–50% of the filament. Optimized formulation was used to print tablets using four different drug models, including 5-ASA, captopril, prednisolone, and theophylline, to show the versatility of their approach [75].

### 3.3. APIs

The choice of the API, or drug, used in the 3D-printed tablet usually determines the other components of the tablet (i.e., excipients and additives) and the suitable AM technology. For instance, filament and melt extrusion-based printing requires elevated temperatures, and APIs that are not thermally stable within the printing temperature range should be avoided. Similarly, SLS utilizes a high-energy laser to sinter the powder formulation, and the APIs should remain stable during this process. SLA requires the use of light (usually in the ultraviolet range) to cure the viscous tablet formulation, and APIs should be stable under light exposure. With this in mind, a wide range of APIs have been used for the AM of oral tablets (Table 1).

Paracetamol and caffeine have been widely used as model drugs in 3D-printed tablets as they are readily available, cost-effective, highly soluble (in water) and permeable. Paracetamol alleviates mild to moderate pain and caffeine is a stimulant to reduce fatigue. For instance, Goyanes et al. used paracetamol as a model drug to extend the release by incorporating different hypromellose acetate succinate (HPMCAS) grades (LG, MG, and HG) into paracetamol-loaded filaments [127]. Sadia et al. used hydrochlorothiazide as a model drug with poor solubility and low permeability to print tablets to accelerate drug release by incorporating built-in channels into their tablet design to increase surface area [74]. Pietrzak et al. selected theophylline as a thermostable model drug, and prepared drug-loaded Eudragit formulations in order to process the tablet formulations at high temperatures (110–170 °C) to eliminate any adverse effects during the HME and FDM printing processes [73].

## 4. Tablet Printing Using AM Technologies

AM technologies have become an attractive option for the fabrication of oral tablets and drug delivery systems. AM technologies, including FDM, DIW, SLS, SLA, DLP, inkjet, and BJ, were successfully used to fabricate tablets with custom-designed shapes and release profiles. AM technologies used with specific polymers and model drugs are summarized in Table 2. Below, we summarize the 3D-printed tablets with respect to the AM technology.

### 4.1. FDM-Printed Tablets

**Figure 2:**
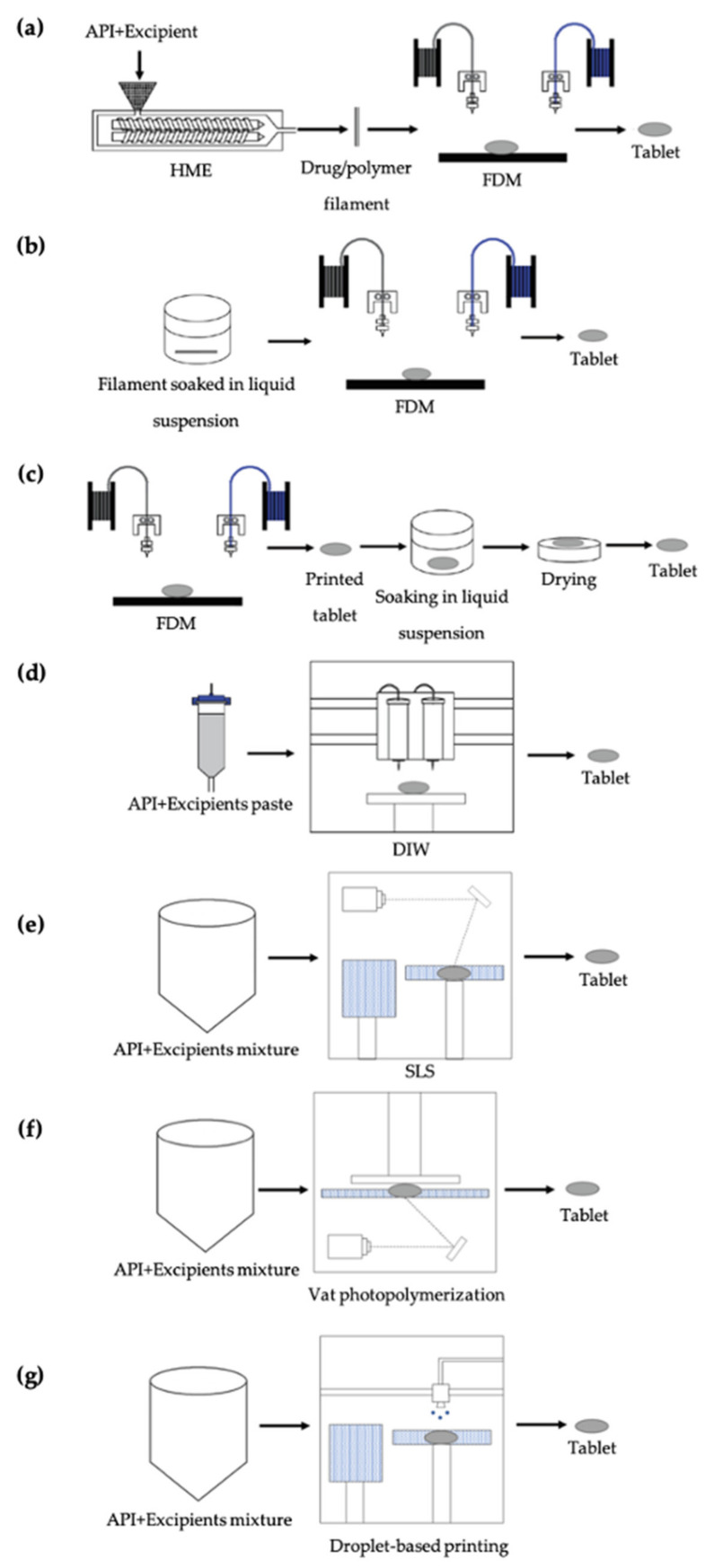
Processes using different 3D printing technologies to produce 3D-printed tablets: (**a**) FDM printing using drug-loaded filaments produced with HME; (**b**) FDM printing with filaments (without APIs); (**c**) FDM printing using drug-infused filaments; (**d**) DIW of drug formulation in the form of a paste; (**e**) SLS printing; (**f**) droplet-based printing; (**g**) vat photopolymerization-based printing.

**Figure 3:**
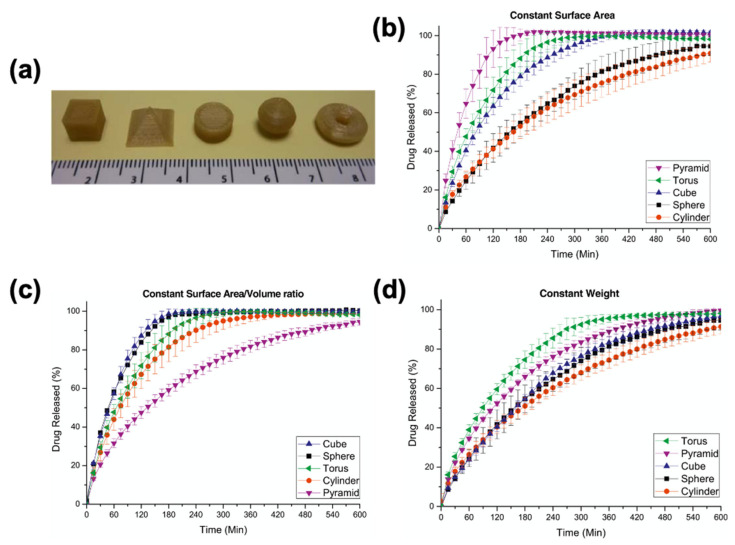
(**a**) FDM-printed paracetamol tablets with different geometries; paracetamol release profile from tablets in phosphate buffer (pH 6.8) with (**b**) 275 mm^2^ surface area; (**c**) surface area to volume ratio of 1; (**d**) 500 mg mass. Adapted with permission from Reference [46]. Copyright 2015, Elsevier.

In tablet printing applications, drug-loaded filaments are commonly utilized with FDM printing technology. APIs containing filaments can be fabricated either by directly incorporating the API into filaments during the HME process (Figure 2a) or by immersing the prefabricated filament in an API solution/suspension to allow diffusion of the API into the filament (Figure 2b) [52,53,54]. It is also possible to incorporate the API directly into the 3D-printed tablets by immersing the tablet into an API solution/suspension (Figure 2c) [96]. Both of the immersion approaches (filament or tablet) are usually time-consuming, require an additional drying process, and allow diffusion of a limited amount of drug [145]. On the other hand, HME allows precise and homogenous incorporation of the API into the filament [146]. Using HME, it is possible to make solid filaments with homogeneous drug dispersion and with good mechanical properties [29,147,148]. Excipients, such as lubricants and plasticizers, in the filament play a major role in the extrusion process. In general, HME allows fabrication of filaments with higher drug amounts and dosage flexibility [11]. Once drug-loaded filaments are fabricated, they can be used directly in FDM printers to print tablets. For instance, Goyanes et al. showed the flexibility of FDM technology to print tablets with different shapes, such as cube, pyramid, cylinder, sphere, and torus, using PVA filaments loaded with paracetamol (Figure 3a) [46]. The authors showed that when the surface area of the printed tablets was constant, drug release was faster in the tablets with a higher surface area to volume ratio, such as a pyramid shape (Figure 3b). When the tablets were printed with a similar surface area to volume ratio, the time needed for 90% drug release were shorter, compared to samples with similar surface areas (Figure 3c). It was also shown that when the tablets were printed with the same weight, drug release profiles were similar to each other (Figure 3d). In another study, Goyanes et al. printed tablets from PVA filaments loaded with different model drugs, including caffeine and paracetamol, at different concentrations [47]. They observed faster drug release for tablets with a higher concentration of drugs. Pietrzak et al. were able to control the release profile of the tablets by simply using different grades of Eudragit filaments loaded with theophylline [73]. In their study, Eudragit E tablets showed an immediate release profile whereas Eudragit RL and Eudragit RS tables showed extended release profiles. Sadia et al. fabricated tablets containing channels of varying number and size (length and width) [74]. The authors showed that inclusion of a larger number of shorter channels accelerates drug release from tablets. Okwuosa et al. utilized PVP in the fabrication of immediate release tablets for the first time [80]. The authors demonstrated the printability of a PVP formulation at relatively low temperatures (110 °C) to show that the usability of FDM can be expanded in tablet printing applications.

In summary, FDM printers allow the fabrication of oral solid dosages with targeted release profiles, e.g., immediate or extended, without additional coating, by simply adjusting the polymer formulation and/or the tablet shape and structure. FDM printing is fast, effective, easy to use and can be used to fabricate tablets with complex shapes. However, the main issue with FDM is that it cannot be used for heat-sensitive APIs or excipients because of possible degradation of the material during the heating process (either during filament fabrication or during the printing process). Due to this limitation, APIs are incorporated into polymer formulations with a relatively lower melting temperature to enable processing and printing at lower temperatures.

### 4.2. DIW-Printed Tablets

**Figure 4:**
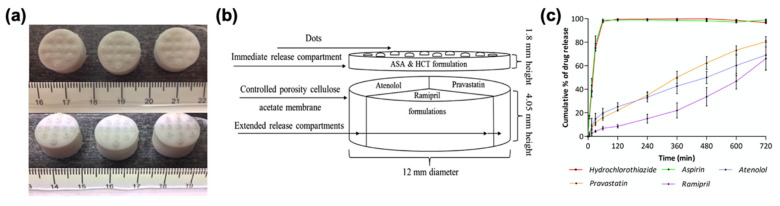
(**a**) DIW-printed tablets with multiple APIs (atenolol, pravastatin, ramipril, aspirin, and hydrochlorothiazide); (**b**) diagram of the tablet design with immediate and extended release compartments; (**c**) drug release profile of each drug from the tablet. Adapted with permission from Reference [138]. Copyright 2015, Elsevier.

Although it is not as popular as FDM in tablet printing, DIW has recently been introduced to the pharmaceutical applications for oral dosage fabrication. To prepare the material to be extruded, excipients and APIs are mixed to obtain a viscous paste. Then the printer cartridge is filled with the paste formulation and tablets are printed by layer-by-layer deposition of the mixture (Figure 2d) [36,59,82,138]. For instance, Khaled et al. used DIW to fabricate complex tablets including immediate and extended release compartments containing multiple APIs (Figure 4a) [138]. For the immediate release compartment, aspirin and hydrochlorothiazide were used as APIs, in combination with a disintegrant, sodium starch glycolate, and a binder, PVP K30. For the extended release compartment, the authors used atenolol, pravastatin, and ramipril as APIs, HPMC 2208 as a hydrophilic matrix, and lactose as filler (Figure 4b). It was shown that two different drug release mechanisms, immediate and extended, could be achieved in one tablet for various APIs (Figure 4c). In another study, Khaled et al. showed that it was possible to use high drug loading, 80 wt% in the dry mixture and 48.48% in the mixture including water, using a paste formulation containing paracetamol (API), PVP and water (binder mixture), and croscarmellose sodium (disintegrant) to fabricate immediate release tablets [59].

DIW is usually performed at room temperature, which is one of the main advantages of this technique, and pose no risk of degradation due to heat (such as in FDM and SLS) or light exposure (such as in SLA and DLP). Additionally, it significantly reduces the need for pre-processing as it allows printing of highly viscous slurries and allows high drug loading, which is an advantage when compared to inkjet printing. Despite these advantages, there is a risk of phase separation within the slurry, which could potentially affect the drug distribution within the printed tablet.

### 4.3. SLS-Printed Tablets

**Figure 5:**
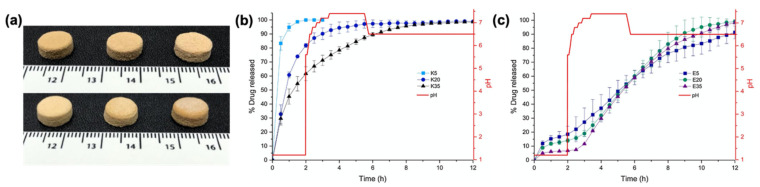
(**a**) SLS-printed paracetamol tablets with different drug loadings (5, 20, 35 and wt%, from left to right). Tablets in the top raw were printed with Kollicoat IR and tablets in the bottom row are printed with Eudragit L100-55; (**b**) drug release profile from tablets printed with Kollicoat IR; (**c**) drug release profile from tablets printed with Eudragit L100-55. Adapted with permission from Reference [109]. Copyright 2017, Elsevier.

In this technology, the API is used as a powder bed and a laser is directed onto the powder bed to sinter the powder spatially (Figure 2e). Powders exposed to laser are bound together following the path of the laser, and this process is repeated layer-by-layer to fabricate 3D tablets. This technology does not use solvents, is a relatively faster manufacturing technology, and allows the fabrication of porous tablets [38,109]. SLS is utilized in two different ways in tablet printing. Drugs can be added before printing or post printing. If the drug is added before printing, a powder mixture of the API and excipients is prepared and added to the powder bed. Then the laser is directed at designed locations to fabricate drug-loaded tablets. When this approach is used, a specific excipient should be added to increase energy absorption and improve printability. For example, Fina et al. added 3% Candurin gold sheen to a powder formulation, which included paracetamol with either Kollicoat IR or Eudragit L100-55, as an absorbent to improve energy absorption [109]. The authors showed that it was possible to use different polymers with various drug concentrations, namely 5%, 20%, and 35 wt%, for SLS printing (Figure 5a). When the printed tablets were tested for dissolution in a dynamic in vitro model, which simulates the pH of the gastrointestinal tract, it was observed that drug dissolution from Kollicoat tablets were pH-independent and tablets with higher drug loading required a longer time for complete drug release due to a less porous tablet matrix (Figure 5b). However, the rate of drug release from Eudragit tablets was pH dependent, as it started to increase at pH > 5.5, and all three drug loading conditions provided similar dissolution profiles (Figure 5c). Salmoria et al. tested the effects of different laser energy densities and particle sizes on tablet morphology and release profile using PCL with progesterone [94]. When a lower particles size was used with higher laser energy density, better sintering was achieved, and when lower laser energy density was used, faster drug dissolution was observed.

Although SLS provides important advantages, such as the immediate usability of drug-mixed powders and the high resolution of the final product, it generally cannot be used with drug-mixed powders, due to the need for high temperatures and high-energy lasers for sintering polymers, which can damage the APIs in the mixture [109]. Since sintering polymers with API is challenging, the drug is incorporated into the tablet post-printing to fabricate drug delivery devices [149,150].

### 4.4. SLA-Printed Tablets

**Figure 6:**
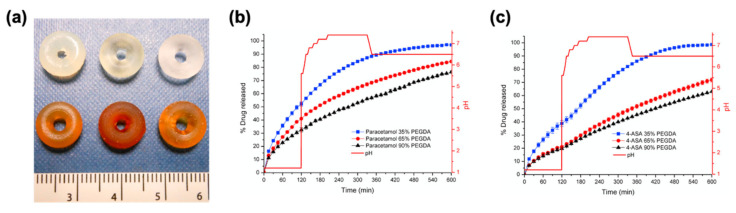
(**a**) SLA-printed paracetamol (top row) and 4-ASA (bottom row) tablets with different photopolymer compositions, from left to right: 35% poly(ethylene glycol) diacrylate (PEGDA)/65% poly(ethylene glycol) 300 (PEG300), 65% PEGDA/35% PEG300, and 90% PEGDA/10% PEG300; (**b**) drug release profile from paracetamol tablets; (**c**) drug release profile from 4-ASA tablets. Adapted with permission from Reference [102]. Copyright 2016, Elsevier.

SLA printers require photocurable solutions (or resins) that photopolymerize or photocrosslink when exposed to a projected laser (Figure 2f). In tablet printing applications, to fabricate drug-loaded tablets, the APIs and excipients should be loaded in the photocurable solution to form a photoreactive solution. This requires use of excess API, as not all of the photocurable solution within the vat is used for printing. In addition, unreacted components can be toxic, which limits the use of SLA technology in tablet printing [151]. SLA usually requires post-processing steps to further cure and/or remove the uncured components. Post-processing approaches include washing with an organic solvent, swelling out the uncured components, exposing the printed construct to light to complete the curing, or heating the printed construct to cure the uncured components. Despite these disadvantages, SLA was shown to fabricate model oral tablets. For instance, Wang et al. prepared a photoreactive solution using PEGDA, PEG 300, and diphenyl(2,4,6-trimethylbenzoyl) phosphine oxide (DPPO) including paracetamol and 4-ASA as a model drug by mixing the components for at least 8 h [102]. The authors showed the suitability of using SLA to print drug-loaded tablets (Figure 6a) with an extended release profile for paracetamol (Figure 6b) and 4-ASA tablets (Figure 6c). In another study, Martinez et al. fabricated ibuprofen-loaded hydrogels using SLA technology [104]. The authors used PEGDA and PEG300, and utilized riboflavin, triethanolamine, and DPPO to induce crosslinking. It was also shown that when water is added to the resin formulation, the drug release rate from the drug-loaded hydrogels could be enhanced.

### 4.5. DLP-Printed Tablets

**Figure 7:**
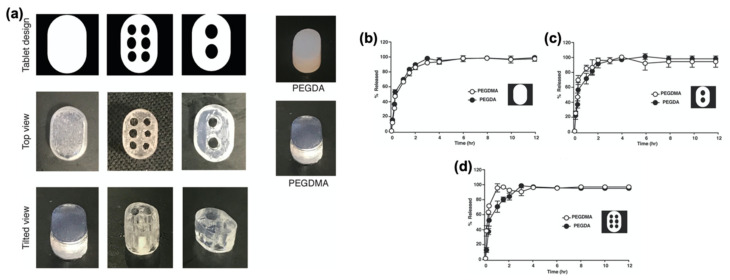
(**a**) DLP-printed theophylline tablets with PEG (polyethlylene glycol) and dimethacrylate (PEGDMA) with different geometries; drug release profiles from tablets with (**b**) no holes; (**c**) two holes; (**d**) six holes. Adapted with permission from Reference [105]. Copyright 2019, Elsevier.

DLP printers have a very similar working principle to SLA printers. They also require a photosensitive material to be cured by exposing the solution to a light source. The main difference between SLA and DLP is how the light source is projected onto the photosensitive resin. In SLA printers, a point laser is used, and resin is cured locally. However, in DLP, since the light is projected onto the resin by a digital projector as two-dimensional patterns, entire layers are cured at once. Due to this advantage, DLP printers are faster than SLA printers in terms of fabricating 3D objects [23,152]. DLP printers provide similar advantages and disadvantages to SLA printers. Kadry et al. used a DLP printer for the first time to fabricate theophylline tablets [105]. The authors used PEGDA and PEGDMA as photoreactive solutions separately to fabricate theophylline tablets with various geometries, including tablets with no holes, two holes, and six holes (Figure 7a). Incorporating holes in the design improved the drug dissolution rate, and it was observed that PEGDMA was a better option for immediate release tablets, as drug release was faster in PEGDMA tablets compared to PEGDA tablets (Figure 7b–d). It was also shown that by adjusting polymer concentration, UV intensity, and UV exposure time, printing conditions could be optimized. Krkobabic et al. used three different hydrophilic excipients, namely PEG 400, sodium chloride, and mannitol, to improve drug release from PEGDA/paracetamol tablets fabricated using a DLP printer [106]. It was shown that when PEGDA content was decreased and PEG 400 content was increased, drug release could be improved, due to a lower degree of crosslinking in the tablets. Although adding sodium chloride improved drug release for most of the formulations, it could not improve it for the formulations with higher PEG 400 content. Incorporating mannitol did not enhance drug release for the first two hours; however, after eight hours, a higher percentage of the drug was released in tablets with higher mannitol content.

### 4.6. Inkjet-Printed Tablets

**Figure 8:**
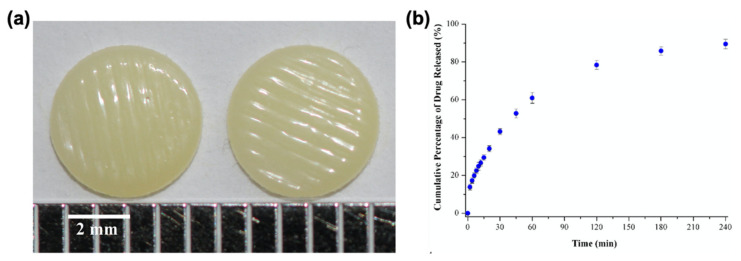
(**a**) Inkjet-printed ropinirole hydrochloride tablets; (**b**) drug release profile in citric acid medium (pH = 4). Adapted with permission from Reference [101]. Copyright 2017, Elsevier.

In inkjet printing technology either photocurable drug-loaded solutions are jetted and crosslinked by a UV light, or drug-loaded polymer melts are jetted and rapidly solidified, after being jetted on the printing platform to fabricate 3D tablets (Figure 2g). Inkjet printing recently started to be used in tablet printing applications. For example, Clark et al. utilized inkjet printing to fabricate tablets using a ropinirole hydrochloride-loaded PEGDA hydrogel matrix crosslinked with Irgacure 2959 (Figure 8a) [101]. The authors showed that the main mechanism for drug release from the highly crosslinked tablets was Fickian diffusion (Figure 8b). Kyobula et al. used hot melt inkjet printing, instead of photocuring of the material, to fabricate honeycomb-like tablets with cell size varying from 0.20 to 1.83 mm [153]. The authors used beeswax as their drug carrier and fenofibrate as the model drug. It was shown that the tablet surface area to volume ratio improved the drug release rate.

Although droplet-based inkjet printing could be successfully utilized for tablet printing applications, the technology possesses some disadvantages. Inkjet printers can only be used with diluted solutions, since the technology allows only employing formulations with low viscosity (<10 mPa·s) [154]. Hence, formulations with high drug loading cannot be used with an inkjet printer. Another characteristic of an inkjet printer is using high shear rates (10^5^–10^6^ s^−1^) to generate droplets [154]. During the printing process, high shear rates can alter APIs [155]. There are also some limitations related to the solution properties. The surface tension values of the formulations used in an inkjet printer determine the droplet formation kinetics. Basically, the shape of the jetted droplet and the droplet on the printing substrate will be affected by the surface energy of the formulation. The surface tension of the formulations used in the technology varies between 28 and 350 mN·m^−1^ [154]. Droplet–substrate interactions [20,21] and interactions between droplets jetted onto the substrate [18,19] define the resolution and accuracy of the printed tablets. In short, the surface energy and shear viscosity of the formulations place some limitations on tablet printing applications.

### 4.7. BJ-Printed Tablets

Binder jetting technology utilizes powder, and liquid binder solution is jetted onto the powder layer to bind them together spatially. The main advantage of binder jetting is the accuracy of deposition of the binder, leading to uniform content. This is the approach used to fabricate the first FDA-approved drug Spritam^®^, a rapidly disintegrating oral tablet containing levetiracetam. These tablets are available in dosages of 250, 500, 750, and 1000 mg [156]. Although using powders directly is an advantage, considering the conventional manufacturing approaches which also utilize powders, BJ creates highly porous structures with low mechanical properties. To reduce porosity and increase mechanical properties, post-processing methods including sintering could be required.

**Figure 9:**
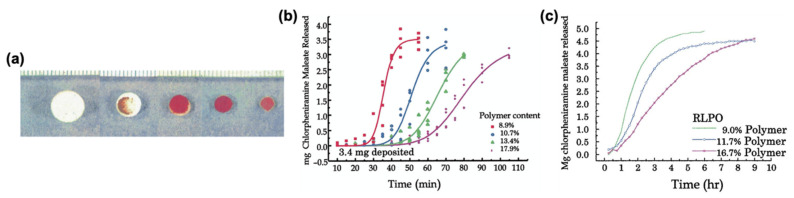
(**a**) BJ-printed Eudragit tablets; drug dissolution profiles of tablets printed with (**b**) Eudragit E-100; (**c**) Eudragit RLPO. Adapted with permission from Reference [77]. Copyright 2000, Elsevier.

Katstra et al. used pharmaceutical-grade cellulose powder with two different binder solutions, Eudragit E-100 and ethanol (Figure 9a), and Eudragit RLPO [77]. The authors showed that increasing polymer content resulted in delayed release profiles of chlorpheniramine maleate in tablets printed with both Eudragit E-100 (Figure 9b) and Eudragit RLPO (Figure 9c). Additionally, the authors showed that the mechanical properties of the tablets were similar to those of commercial (compressed) tablets [77]. Rowe et al. fabricated four different complex oral dosage forms, including an immediate-extended release device, a breakaway tablet, an enteric dual pulse release device, and a pulsatory release tablet, to show the potential of 3D printing technologies in complex tablet fabrication with tunable release profiles [78]. It was shown that two different release mechanisms, erosion and diffusion, could be incorporated in one device, and pulsatory devices could be fabricated to release one pulse in the stomach and the second in the intestine.

## 5. Challenges and Potential Strategies

Despite being a promising method for the fabrication of oral tablets, AM faces a number of challenges in the pharmaceutical industry, and it is fair to say that it is still at the development stage. These challenges arise from the limitations of the printing technologies and the lack of printable drug formulations. General printing challenges in tablet printing applications include inconsistent layer thickness, insufficient adhesion between consecutive layers, inconsistent print patterns (overall shape, porosity, mechanical properties), and obtaining friable tablets (with a tendency to chop, crumble, or break during compression). These issues result in quality issues in the final product, such as undesired tablet dimension and shape, or even unsuccessful print due to collapsing of the print. While some of these challenges arise from issues with software and hardware deficiencies, such as inconsistent print patterns, most of them arise from material properties of the formulation used. To ensure high-quality tablet fabrication, material properties, such as drug concentration, and use of proper excipients and polymers, need to be optimized according to the AM technology used. Additionally, it is crucial to optimize printing parameters such as infill density, extrusion speed, temperature, and pressure to improve the quality of the final product [157]. 3D processing of drugs is different than the conventional compression method and further understanding of the APIs is necessary, including their solubility, stability, crystallinity, and thermal stability, to utilize them in the printing process [158]. Another crucial factor in pharmaceutical production is to maintain reproducibility. When a melt-based, a paste-based, or a solution-based ink is used for tablet printing, it is important to maintain the homogeneity of the ink formulation. During a prolonged period of use, the ink could phase-separate, leading to the printing of tablets with inconsistent compositions. Note that this could happen to the ink in the print head (extrusion-based printing, inkjet printing, or BJ) or in the vat (SLA or DLP). This could also result in clogging inside the print head nozzle for extrusion and droplet-based AM technologies.

Each available AM technology has its own advantages and limitations in tablet fabrication processes (Table 3), mainly due to the material requirements and processing conditions. FDM is the most widely used AM technology in tablet printing studies. As discussed in the previous sections, the required form of the material in FDM printing is filaments, which are generally fabricated with HME. This limits the usable materials to non-heat sensitive APIs and excipients, and thermoplastic polymers, which may not be pharmaceutically approved [159]. When drugs are directly loaded into the filaments by a soaking method (diffusion), it is not possible to achieve high drug loadings [145]. Another extrusion-based technology used in tablet printing applications is DIW, which only allows viscous formulations in paste form. Note that it is also possible to directly melt the powder formulation within the DIW printhead, and print the melt, yet prolonged exposure of the polymer leads to degradation. As discussed earlier, this technology generally operates at room temperature when paste is used, which removes the risk of thermal degradation of APIs in the formulation; however, there is a risk of phase separation and it can be challenging to achieve uniform drug loading within the tablet. Although powder-based printing technologies, such as SLS, allow direct use of pharmaceutical powder formulations, the main issue with this technology is the potential degradation or deterioration of APIs due to the projected high-energy laser onto the drug-loaded formulations. When vat photopolymerization-based printing (SLA and DLP) is used, tablet formulations need to be in the form of a photocurable viscous solution, which significantly limits the available material systems. In inkjet printing applications, it is challenging to use high drug loading, due to the need for low viscosity formulations, and exposure to high shear during the printing process could lead to shear-induced deterioration of the APIs. Finally, in BJ, generally highly porous tablets are fabricated, and their mechanical properties are relatively lower without post-processing.

Tablets printed with AM technologies usually require post-processing, such as drying, sintering, light curing, or removal of the support material, which could prolong the fabrication process and lead to deterioration of the APIs. These post-processing steps are generally required to maintain the shape of the final product or to improve material properties, such as mechanical properties. The most common post-printing process for all AM technologies is removal of the support material (except for powder-based printing). Although it is not always required to print support material, it might be required to ensure successful tablet fabrication. This support material should be removed after printing and the tablet should be handled carefully and not be damaged during this process. The other post-processing methods are generally specific to the selected AM technology. FDM generally does not require additional post-processing methods, which makes them a favorable option for the rapid fabrication of tablets. DIW printing technology requires some additional drying process after printing to ensure that the paste is dried and the tablet is structurally stable. This process can take a few hours [59]. In vat photopolymerization-based printing technologies (SLA and DLP), post-processing includes a washing step to remove unreacted chemicals, and if needed swelling out the uncured chemicals, prolonged light exposure, or heat treatment, to ensure complete curing. In inkjet printing, a drying process is needed, and if a photocurable ink is used prolonged light exposure or a heat treatment step is required. For powder-based printing approaches, a heat treatment step is required to reduce porosity and increase the mechanical properties of the constructs.

Another issue is the lack of quality control processes and regulations for the AM of oral tablets [13,38,160]. Quality control processes are crucial to ensure tablet quality and reproducibility. As discussed above, the general printing challenges need to be addressed by in-line quality control strategies. These could include real-time monitoring for print pattern and layer thickness by using a high-resolution video camera that could be run together with software to enable real-time manipulation of the printing process (i.e., the g-code). Another strategy is real-time testing, such as incorporating in-line sensors to monitor and adjust printing conditions during the printing process. For instance, a nondestructive infrared (IR) camera was commonly used to monitor the evolution of material temperature during FDM [41,42,43,45] and SLS [31,40] printing. Coogan and Kazmer developed in-line rheological monitoring for FDM [161], which could then be used in conjunction with real-time modeling to predict the interlayer strength of the printed constructs [162]. This approach could potentially be applied to SLA and DLP. Regulatory bodies (such as FDA) have instructions and supervision for all of the methods, processes, equipment, and ingredients used in pharmaceutical products. It is still unclear how should regulations be adjusted for AM. Since traditional clearance routes are not well-defined for 3D-printed tablets, there is a need for guidance from regulatory bodies that would define a clear pathway for obtaining regulatory approvals for 3D-printed tablets [13,163].

Considering on-demand manufacturing, it is also imperative to develop standards for training and certification of operators to ensure quality control. 3D printing allows the fabrication of personalized, made on demand drug products that could be printed by healthcare providers based on patients’ needs [164]. Pharmacies and hospitals should have the appropriate environment for tablet production to avoid any contamination. In addition, pharmaceutical ink formulations should be readily available in different dosages. Finally, CAD designs used in tablet production should be modeled with pre-determined release profiles to ensure that the targeted release of the drug can be achieved. These issues create challenges for pharmaceutical companies to switch from mass production of oral solid dosages to production of pharmaceutical ink formulations for individualized production.

## 6. Conclusions

This review article provides a detailed overview of 3D-printed oral tablets with respect to the AM technology. Currently available AM technologies for oral tablet production include extrusion-based FDM and DIW, powder-based SLS, vat photopolymerization-based SLA and DLP, and droplet-based inkjet and BJ. The printability and form (e.g., filament, powder, slurry, viscous liquid, photocurable solution) of a pharmaceutical ink (i.e., an oral tablet formulation) are strictly determined by the specific AM technology. A number of innovative tablets with compositional and architectural complexity have been fabricated using AM technologies leading to custom-designed tablets with user-defined release profiles. Although AM enables the fabrication of custom-designed and personalized oral tablets with a strong potential to move the pharmaceutical industry from mass production to personalized medicine, AM is far from becoming a mainstream manufacturing approach for the fabrication of oral solid dosage forms. This is mainly due to limitations of the AM technologies and a lack of printable drug formulations as well as the lack of a clear path to ensure quality control of printed tablets and to eliminate regulatory issues.

## Figures and Tables

**Table 1:** Different active pharmaceutical ingredients (APIs) used to research 3D printing in the pharmaceutical industry and their effect on the body.

Drug	Effect on the Body	Reference
4-ASA (4-Aminosalicylic acid)	Antibiotic primarily used to treat tuberculosis	[52]
5-ASA (5-aminosalicylic acid or Mesalamine)	Anti-inflammatory	[137]
Aripiprazole	Antipsychotic	[49]
Aspirin	Reduces risk of blood clotting and reduces the risk of heart attacks and strokes	[138]
Atenolol	Used to treat hypertension and prevent heart attack	[138]
Budesonide	Treats inflammatory bowel disease	[139]
Caffeine	Stimulant to reduce fatigue	[45]
Captopril	Lowers blood pressure (for hypertension)	[140]
Deflazacort	Immunosuppressant and anti-inflammatory	[141]
Domperidone	Treats gastroparesis and other conditions causing chronic nausea and vomiting	[142]
Hydrochlorothiazide	Prevents absorption of too much salt and treats oedema	[138]
Paracetamol	Analgesic and Antipyretic	[59]
Pravastatin	Reduces blood cholesterol and triglyceride in hyperlipidemic patients	[138]
Prednisolone	Anti-inflammatory	[143]
Ramipril	Angiotensin (increases blood pressure)	[138]
Theophylline	Bronchodilator	[144]

**Table 2:** Research done on oral tablet printing using various polymers and 3D printing technologies.

Printing Technology	Polymer	Model Drug	Reference
FDM of hot melt extruded loaded filament	EC	Quinine	[30]
		Carbamazepine	[33]
	HPC	Paracetamol	[31]
		Itraconazole	[32]
		Carbamazepine	[33]
		Domperidone	[34]
		Theophylline	[35,73]
	PVA	Paracetamol	[45,46,47]
		Caffeine	[45,47]
		Budesonide	[48]
		Aripiprazole	[49]
		Glipizide	[50]
		Hydrochlorothiazide	[51]
	Eudragit	Hydrochlorothiazide	[74]
		Theophylline	[73,76]
		5-ASA	[75]
		Captopril	[75]
		Prednisolone	[75]
	PVP	Theophylline	[80]
		Dipyridamole	[80]
		Pantoprazole sodium	[81]
FDM, API incorporated by soaking	PVA	4-ASA	[52]
		5-ASA	[52]
		Prednisolone	[53]
		Curcumin	[54]
		Fluorescein	[55]
	Eudragit	Deflazacort	[96]
	PCL	Deflazacort	[96]
DIW	HPMC	Atenolol	[138]
		Pravastatin	[138]
		Ramipril	[138]
		Guaifenesin	[36]
		Dipyridamole	[37]
	PVP	Aspirin	[138]
		Hydrochlorothiazide	[138]
		Paracetamol	[59,82]
	Carbopol	Guaifenesin	[36]
Binder jetting	Eudragit	Chlorpheniramine maleate	[77,78]
	PVP	Paracetamol	[83,136]
Inkjet printing	PEGDA	Ropinirole hydrochloride	[101]
SLS	PCL	Progesterone	[94,95]
SLA	PEGDA	Paracetamol	[102,103]
		4-ASA	[102]
		Aspirin	[103]
		Ibuprofen	[104]
DLP	PEGDA	Theophylline	[105]
		Paracetamol	[106]
	PEGDMA	Theophylline	[105]

**Table 3:** Advantages and disadvantages of printing technologies used in tablet printing applications.

Printing Technology	Advantages	Disadvantages
FDM	High drug loading Can print complex shapes Easy to adjust drug release profiles	Preprinting processes can take time Possible thermal degradation of APIs
DIW	High drug loading No risk of thermal degradation	Possible phase separation of drug formulations Hard to uniformly distribute APIs within the paste Drying of the tablet is required post printing
SLS	High resolution No need for preprinting processes	Possible degradation of APIs due to sintering
SLA	High resolution	Possible degradation of APIs due to laser projected onto the drug-loaded solution Additional post-printing processes needed, such as photocuring of the final product
DLP	High resolution	Possible degradation of APIs due to laser projected onto the drug-loaded solution Additional post-printing processes needed, such as photocuring of the final product
Inkjet	Versatility of the technology, can be used with heat or light-based approach	Cannot be used with high drug loading APIs can be affected by high shear rates during printing
BJ	Uniform final product	Highly porous final products lead to low mechanical properties Requires additional post-printing processes such as sintering

## Data Availability

Not applicable.
